# Microwave-Based Colonoscopy: Preclinical Evaluation in an Ex Vivo Human Colon Model

**DOI:** 10.1155/2022/9522737

**Published:** 2022-01-28

**Authors:** Glòria Fernández-Esparrach, Alejandra Garrido, Roberto Sont, Walid Dghoughi, Sergi Marcoval, Miriam Cuatrecasas, Sandra López-Prades, F. Borja de Lacy, María Pellisé, Ignasi Belda, Marta Guardiola

**Affiliations:** ^1^Endoscopy Unit, Gastroenterology Department, Hospital Clínic, University of Barcelona, CIBEREHD IDIBAPS, Barcelona, Spain; ^2^MiWEndo Solutions, Barcelona, Spain; ^3^Pathology Department, Hospital Clínic, University of Barcelona, CIBEREHD, IDIBAPS, Spain; ^4^Gastrointestinal Surgery Department, Hospital Clinic, University of Barcelona, Spain

## Abstract

**Introduction:**

Microwave imaging can obtain 360° anatomical and functional images of the colon representing the existing contrast in dielectric properties between different tissues. Microwaves are safe (nonionizing) and have the potential of reducing the visualization problems of conventional colonoscopy. This study assessed the efficacy of a microwave-based colonoscopy device to detect neoplastic lesions in an ex vivo human colon model.

**Methods:**

Fresh surgically excised colorectal specimens containing cancer or polyps were fixed to a 3D positioning system, and the accessory device was introduced horizontally inside the ex vivo colon lumen and moved along it simulating a real colonoscopy exploration. Measurements of the colon were taken every 4 mm with the microwave-based colonoscopy device and processed with a microwave imaging algorithm.

**Results:**

14 ex vivo human colorectal specimens with carcinomas (*n* = 11) or adenomas with high grade dysplasia (*n* = 3) were examined with a microwave-based device. Using a detection threshold of 2.79 for the dielectric property contrast, all lesions were detected without false positives or false negatives.

**Conclusions:**

This study demonstrates the use of a microwave-based device to be used as an accessory of a standard colonoscope to detect neoplastic lesions in surgically excised colorectal specimens.

## 1. Introduction

To date, colonoscopy is the most effective diagnostic and therapeutic technique for the prevention of colorectal cancer (CRC), since it allows the identification and removal of polyps with a relatively good accuracy. Several prospective studies demonstrate that colonoscopy with polypectomy reduces the incidence of CRC by 40–90% [1, 2].

Nevertheless, colonoscopy is far from being perfect: 22% of polyps are not detected [3], and the risk of cancer after a negative colonoscopy is still 7.9% [4]. The main cause of this lack of efficacy is the visualization limitation [5] of the optical camera placed at the tip of the endoscope. Studies indicate that 13.4% of the colon surface area might not be visualized during a standard colonoscopy [6] due to reduced field of vision (<180°), inhomogeneous illumination, colon angulation and folds, and poor cleaning. In recent years, several devices and technologies have been developed to improve the detection rate of polyps, such as high-definition endoscopes, endoscopes with multiple lenses (retrovision capability), and mucosal flattening accessories [7].

Microwave imaging can obtain anatomical and functional images of the interior of the human body representing the existing contrast in dielectric properties between different tissues. Microwaves can generate images without restriction of the field of view (360°), are safe (nonionizing and without thermal effect), and offer a fair trade-off between resolution and light opaque tissue penetration [8, 9], therefore, potentially reducing visualization problems of conventional colonoscopy. We recently demonstrated that the dielectric properties correlate with the malignancy and grade of dysplasia of colorectal polyps [10]. For these reasons, microwave imaging has the potential to complement conventional colonoscopy to improve both polyp detection rate and in situ tissue classification.

This study assessed the potential of microwave-based colonoscopy to detect neoplastic lesions in an ex vivo human colorectal model.

## 2. Materials and Methods

### 2.1. Microwave-Based Accessory Device Description

**Figure 1:**
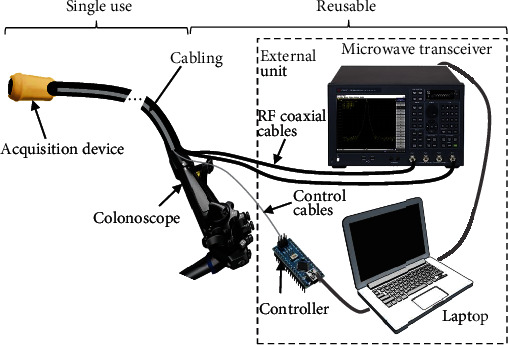
The imaging system consists of a cylindrical ring-shaped acquisition device attached to the tip of a colonoscope connected via cables to the external unit. The external unit consists of a microwave transceiver, a microcontroller, and a laptop.

The imaging system consists of (1) a cylindrical ring-shaped acquisition device that can be attached to the tip of a conventional colonoscope; and (2) an external unit with a microwave transceiver, a controlling unit, and a laptop as a processing unit. The acquisition device contains two switched arrays of eight antennas organized in two rings, one containing the transmitters and the other the receivers [11] that are encapsulated and are connected via cables to the external unit (Figure 1). The dimensions of the acquisition device are 30 mm in length by 20 mm in diameter, having a total thickness of 2.5 mm. The dimensions and shape of the device ensure nonobstruction of the front tip of the colonoscope, avoiding camera concealment, injuring the patient or hindering the maneuverability of the colonoscope.

### 2.2. Microwave-Based Colonoscopy Setup for Ex Vivo Colon Measurements

**Figure 2:**
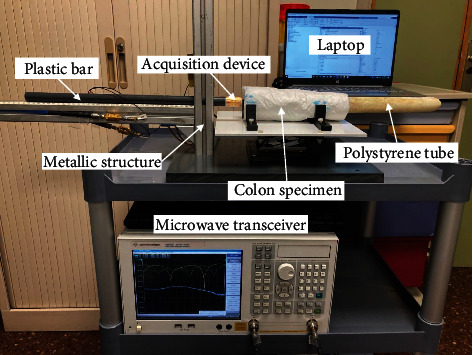
Measurement setup. The colon is wrapped around a polystyrene tube, and the acquisition device is attached to the tip of a plastic bar and connected to the microwave transceiver, the microcontroller, and the laptop.

To be able to reproduce a real colonoscopy exploration, a 3D positioning system was built (Figure 2) as described elsewhere [12]. The measurement setup is composed by an L-shaped metallic structure fixed on a plastic base. The metallic structure holds a plastic bar that simulates a colonoscope, and the acquisition device is attached at the tip. With this bar, the accessory device was introduced horizontally inside the ex vivo colon or rectal lumen and was moved along it to obtain the measurements.

Surgically excised colorectal specimens containing cancer or polyps were used for the measurements. Immediately after the excision, the fresh specimens were opened longitudinally and then fixed around a tube made of expanded polystyrene and wrapped with a soaker pad. Expanded polystyrene behaves like air for microwaves and therefore does not interfere with measurements. After the procedure, the specimens were sent to the Pathology Department for tissue processing and histological analysis. The protocol was approved by the Ethics Committee of Hospital Clinic of Barcelona, and patients gave written informed consent.

### 2.3. Microwave Imaging Method for Colonoscopy Description

Microwave imaging is based on illuminating the colon with a microwave signal emanating from one transmitting antenna. The total received field, resulting from the interaction of the incident microwaves, and the colorectal tissues are then measured at the receiving antenna adjacent to the active transmitting antenna. The previous process is repeated for each transmitting antenna located around the tip of the colonoscope, in this way, information is obtained from the entire perimeter of the colon or rectum. The received field contains information of the spatial changes of the dielectric properties of the tissues. By processing the total field with an imaging algorithm, the dielectric property contrast of the colorectal tissues can be retrieved. The information obtained represents a cross-sectional slice of the colon or rectum, which we call a frame. As the colonoscope moves, the acquisition device is continuously scanning frames, thus, covering all colorectal lumen surface.

## 3. Results

**Figure 3:**
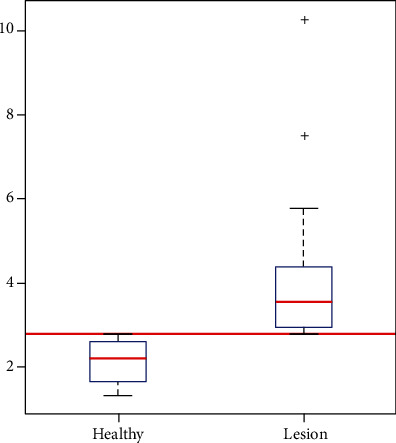
Reconstructed maximum dielectric property contrast of each patient and frame obtained with microwave-based colonoscopy for healthy mucosa areas and for lesions. The detection threshold fixed to 2.79 is represented as a horizontal red line. All values above the line are classified as lesion and below as healthy mucosa.

Fourteen colorectal surgical specimens containing carcinomas (*n* = 11) or adenomas with high grade dysplasia (*n* = 3) were examined (Table 1). The median dielectric property contrast was 3.56 (range, 2.79-10.28) for neoplastic lesions and 2.20 (range 1.31-2.78) for normal mucosa. Setting the dielectric contrast threshold at 2.79, all lesions were detected without false positives or false negatives (Figure 3).

**Figure 4:**
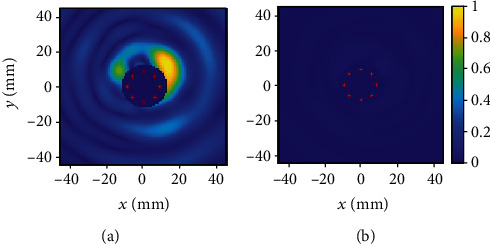
Image frames obtained with microwave-based colonoscopy. The dark blue circle in the middle of the plot indicates the position of the acquisition device, and the red dots the position of each antenna. (a) The highest intensity yellow spot indicates the presence of the polyp. (b) The colors are uniform showing that there are no lesions in the mucosa.

**Figure 5:**
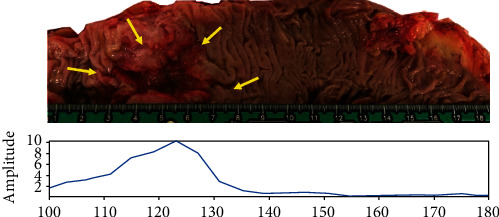
Correlation with the evolution of the maximum amplitude of each successive frame obtained with microwave-based colonoscopy (bottom) and the location of the lesion indicated by the arrows (top). Lower amplitudes around 0.2 are typical of healthy mucosa areas, while neoplastic lesions show high amplitudes close to 1.

The results of the microwave-based colonoscopy can also be represented as an image (Figure 4). The evolution of the measurements in each frame and the correspondence with the location of the lesion are shown in Figure 5.

## 4. Discussion

In this study, we have demonstrated for the first time the feasibility of using microwaves for diagnosing colorectal neoplasms. To do so, an accessory device attachable at the distal end of a conventional colonoscope has been developed and tested on human ex vivo colorectal surgical specimens with neoplasms.

Missing lesions at colonoscopy are as high as 22% and have important clinical consequences. In the last years, many efforts have been made to improve the performance of endoscopy, mainly based on improvements in the image quality and a better inspection of the mucosa. However, all these technologies (virtual chromoendoscopy, devices that flatten the mucosa, endoscope with retrovision capability…) cannot see what is not captured in the image. Contrarily, our microwave-based device can differentiate between healthy mucosa and neoplastic lesions based on the changes in their dielectric properties and complementing the endoscopic image emitting an alarm when the dielectric contrast is higher than a predefined threshold. The potential of microwave-based colonoscopy is especially relevant in small flat adenomas which constitute the majority of missed lesions. In a previous study, we did not find significant differences in dielectric properties due to the shape of the polyps [10].

The system has been designed to be compatible with colonoscopy, ensure a 360° coverage, and produce minimal changes to the current clinical practice. Concerning the size restrictions, the final dimensions of the acquisition device are 30 mm in length by 20 mm in diameter, having a total thickness of 2.5 mm. The dimensions and shape of the device ensure nonobstruction of the front tip of the colonoscope and avoid injuring the patient or hindering the maneuverability of the colonoscope. The device has recently been verified on a colon phantom that simulates a realistic colonoscopy exploration. The phantom models a section of a colon including the haustrum and allows placement of different polyps. The phantom is composed of gelatin-oil-based materials to mimic the dielectric properties of colon healthy mucosa and polyps with high grade dysplasia [12].

We used the IDEAL model to drive this innovation [13–15]. The IDEAL Framework and Recommendations represent a new paradigm for the evaluation of surgical operations, invasive medical devices, and other complex therapeutic interventions. The 2009 IDEAL comprised 5 stages: idea (1), development (2a), exploration (2b), assessment (3), and long-term study (4). The original framework was updated in 2019, and a stage 0 was considered, which includes preclinical studies with material testing, simulator, cadavers, animal, and modeling, among others. Preclinical studies are of paramount importance to advance in the conceptualization and preparation before its implementation in patients.

The main limitation of this study is the low number of adenomas studied and reflects the lack of anatomic models provided with haustral folds and polyps made of materials that have the same dielectric properties as the real cases. Therefore, we had to assess our device in ex vivo human colorectal specimens that usually are surgically resected because they have already developed cancer. However, since the grade of dysplasia can be inferred from the dielectric properties as they increase with dysplasia [10], microwave-based colonoscopy has the potential of diagnosing adenomas with low grade dysplasia as well. The second limitation is that measurements were performed in tissues without vascularization. Since the contrast in dielectric properties between different tissues depends on the amount of water, the contrast threshold could be different in an in vivo experiment. The last limitation is that the device was not attached at the end of a real endoscope, and the maneuvrability could not be assessed. We are planning new studies in animal models to check the maneuvrability and intercompatibility with other endoscopic devices, including those that use electrocautery.

In summary, this study demonstrates for the first time the use of a microwave-based device to be used as an accessory of a standard colonoscope to detect neoplastic lesions in surgically excised colorectal specimens.

## Figures and Tables

**Table 1:** Histology type and size of the lesions.

Patient	Age	Histology type of the lesion	Size (mm)
1	86	Adenoma with HGD	10
2	64	Adenocarcinoma	50
3	46	Adenocarcinoma	36
4	37	Adenoma with HGD	32
5	83	Adenocarcinoma	48
6	60	Adenocarcinoma	37
7	57	Adenocarcinoma	65
8	68	Adenocarcinoma	15
9	86	Adenoma with HGD	23
10	85	Adenocarcinoma	34
11	45	Adenocarcinoma	32
12	91	Adenocarcinoma	40
13	62	Adenocarcinoma	37
14	81	Adenocarcinoma	63

HGD: high grade dysplasia.

## Data Availability

All data generated or analysed during this study are included in this article. Further enquiries can be directed to the corresponding author.
